# Lisfranc Sprain with Second Metatarsal Base Adaptive Stress Response in High-Level Athletes: Case Series and Novel Perspective on a Distinct Entity of Chronic Low-Energy Lisfranc Injury

**DOI:** 10.1155/2022/1030829

**Published:** 2022-02-10

**Authors:** WanYin Lim, Jonathan Heysen, James Ilic, Ben Beamond, Steven Zadow

**Affiliations:** ^1^Dr Jones and Partners Medical Imaging, 226 Greenhill Road, Eastwood, South Australia 5063, Australia; ^2^Radiology Department, Royal Adelaide Hospital, 1 Port Rd, South Australia 5000, Australia; ^3^Wakefield Sports and Exercise Medicine Clinic, Calvary Adelaide Hospital, South Australia, Australia; ^4^Wakefield Orthopaedic Clinic, Calvary Adelaide Hospital, South Australia, Australia; ^5^Flinders Medical Centre, South Australia, Australia

## Abstract

Lisfranc injury is increasingly being recognised in the high-performance athletic cohort, particularly in contact sports. In this cohort, there is a pattern of low-energy Lisfranc injury which combines magnetic resonance findings of both ligamentous sprain and adaptive bone stress response that infers a longer timeframe of stress than the duration of symptoms would suggest. This has not been previously described, and the authors believe that this is an unrecognized subset of midfoot sprain in the context of sustained stress to the midfoot. This retrospective case report describes MRI findings of three index cases of this entity in professional athletes presenting with acute foot pain. Two responded with conservative management whilst the third ultimately required surgery. All athletes were eventually able to return to play.

## 1. Introduction

Foot injury accounts for 16% of all sports-related injuries of which low-energy Lisfranc injury is uncommon but increasingly being diagnosed, presumably from the increased awareness of this condition and the increased use of magnetic resonance imaging (MRI) in imaging of foot pain [1–5]. In contact sports such as soccer and American football where running, jumping, and twisting of the foot in plantarflexion are common, the Lisfranc injury incidence can be as high as 1.9-4% per year [5–7]. This pattern of injury is quoted to have a misdiagnosis rate of up to 39% at first presentation, although the actual prevalence or rate of underdiagnosis is unknown, with significant long-term consequences which include career-ending morbidity [7].

In elite international athletes that we have imaged in the acute setting, we have noticed a pattern of Lisfranc injury that has not been previously described, which includes a mixed picture of acute Lisfranc sprain and chronic adaptive response of the Lisfranc bone unit. It has also come to the authors' attention that there is a relative paucity in radiology literature on Lisfranc ligamentous injury in athletes with low-energy Lisfranc injuries.

This manuscript showcases three high-level athletes with Lisfranc ligament injury demonstrating chronic stress response in the metatarsal bases. It provides a hypothesis for the imaging findings.

## 2. Case Presentation

This is a retrospective study from January 2019 to June 2020. Three international athletes who presented to the radiology clinic for investigation of acute-onset foot pain were identified. All the studies were reported by dedicated musculoskeletal radiologists with combined experience of 45 years.

### 2.1. Case 1

A 19-year-old international-level male soccer player presents with a two-week history of midfoot pain, worse with running. This occurred during an overseas match. There was no single traumatic event recalled. There was no other medical comorbidity. Routine panel of metabolic and blood screen including calcium and vitamin-D level was normal at the start of the season.

**Figure 1:**
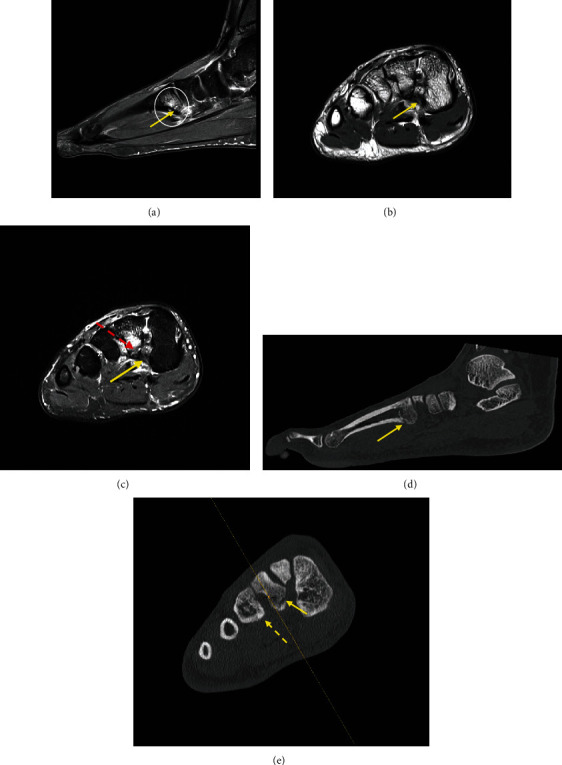
(a) Sagittal proton-density-weighted, fat-suppressed sequence (PDFS) at the level of the second metatarsal. There is localized marrow oedema signal centered at the base of the second metatarsal plantar margin (circled), with adjacent soft tissue oedema. The inferior cortex is thickened, with presence of intracortical high signal consistent with osteitis (arrow). Periosteal oedema present. The intracortical signal traversing the base of the second metatarsal is suspicious for an undisplaced fracture. Axial sequences at the level of the second metatarsal base in proton density (PD) (b) and PDFS (c) show thickening of the plantar Lisfranc ligament (arrow) which returns intermediate signal but is otherwise intact, consistent with grade II sprain or partial tear. Undisplaced fracture at the metatarsal base as shown (dashed arrow). Sagittal (d) and axial (e) CT at the similar level demonstrates the avulsion fracture at the base of the second metatarsal at the site of the plantar Lisfranc ligament insertion (arrow). There is also cortical thickening of the border of the third metatarsal base at the site of plantar Lisfranc attachment (dashed arrow).

The MRI showed localized marrow oedema signal centered at the base of the second metatarsal with suspicion of an avulsion fracture at the footprint of the plantar Lisfranc ligament (Figure 1(a)). The cortex of the metatarsal base was thickened, with intracortical fluid-hyperintense signal present, consistent with chronic stress response. The Lisfranc ligament was thickened and returns intermediate signal with surrounding soft tissue oedema, compatible with a grade II sprain or partial tear (Figures 1(b) and 1(c)).

The undisplaced avulsion fracture was confirmed on computed tomography (CT) (Figures 1(d) and 1(e)).

He was treated conservatively with protected weight-bearing for 6 weeks with good outcome and was able to return to baseline shortly after.

### 2.2. Case 2

An 18-year-old male Australian footballer presents with sudden-onset midfoot pain, exacerbated by running and jumping.

**Figure 2:**
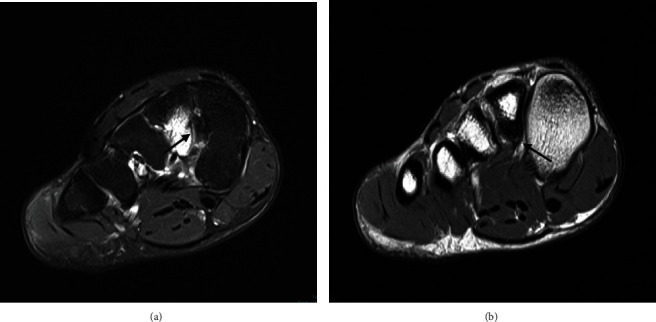
(a, b) An 18-year-old footballer with second metatarsal tenderness worse on running and jumping. Axial PDFS (a) shows localized marrow oedema signal at the plantar margin of the base of the second metatarsal involving both interosseous (arrow) and plantar Lisfranc footprints (dash). Minor Lisfranc proper periligamentous oedema without a discrete tear or elongation identified, consistent with grade I sprain. Axial PD (b) clearly demonstrates the cortical thickening at the second metatarsal base (arrow).

The MRI demonstrated localized marrow oedema signal at the plantar margin of the second metatarsal base (Figure 2(a)). This involved both the interosseous and plantar Lisfranc footprints. There was periligamentous oedema without a ligamentous fiber disruption or elongation, consistent with grade I sprain (Figure 2(a)).

There was second metatarsal cortical thickening centered around the ligamentous attachments (Figure 2(b)). This was also treated conservatively, with return to play after 3 months.

### 2.3. Case 3

A 29-year-old female elite soccer player presents with nonspecific, 1-day history of foot pain during an international soccer tournament. She was unable to complete warmup. There was no history of trauma recalled. There is no significant past medical history apart from mild asthma.

On assessment, she had an antalgic gait.

**Figure 3:**
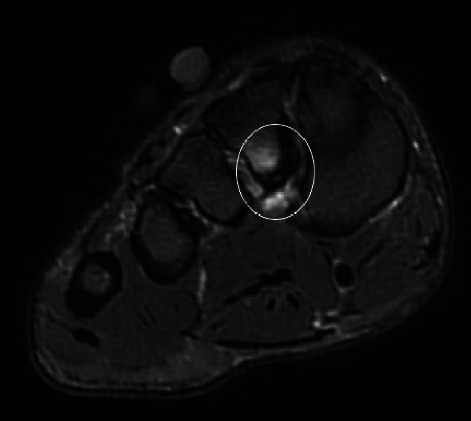
A 29-year-old soccer player with progressive midfoot pain. Axial PDFS at the plantar Lisfranc attachment to the second metatarsal base. There is oedema signal associated with the plantar Lisfranc ligament. There is also plantar cortical thickening with intracortical fluid signal (circled).

On imaging, there was periligamentous oedema surrounding the otherwise-intact plantar Lisfranc ligament (Figure 3). Plantar cortical thickening was indicative of adaptive response. Intracortical fluid hyperintensity was also present, suggesting progression to a higher-grade stress response and impending fracture.

Patient had to be taken out of the soccer tournament. Following a 3-month period of failed conservative management, this was surgically managed as a nonhealing stress fracture with drilling and plate fixation of the second metatarsal base. She responded well to treatment and was able to return to game at prior baseline level.

## 3. Discussion

The Lisfranc ligamentous complex confers stability to the tarsometatarsal (TMT) articulation by providing structural support to the transverse arch of the mid- and forefoot. The first intermetatarsal space is held by the dorsal, interosseous, and plantar ligaments extending from the medial cuneiform to the base of the second metatarsal. These three ligaments constitute the Lisfranc proper [4, 8]. The interosseous ligament connects the medial cuneiform to the base of the second metatarsal whilst the plantar Lisfranc ligament connects the medial cuneiform to the base of second and third metatarsals. These two are considered the strongest ligaments in the Lisfranc ligamentous complex and are the primary stabilizer to the medial and middle column of the midfoot [4, 8]. This arrangement allows the foot to perform as a lever arm that propels the foot forward during the gait cycle [9].

Injuries to this Lisfranc complex can be differentiated into high-impact or low-impact trauma. The low-impact trauma, also coined midfoot sprain, constitutes up to a third of Lisfranc injuries and will be the main focus of this manuscript [10]. These are usually a result of indirect forces applied to the foot when the foot is in certain fixed positions. The most common mechanism of injury is when the forefoot is abducted and plantarflexed in a fixed position, such as in the context of sudden rotational change in the direction that occurs during tackling. This results in the weight of the body being placed on the tarsometatarsal joints, resulting in ligamentous failure. More commonly, in sports requiring cleated shoe wear, the mechanism for the injury is from rigidly planted forefoot in a plantarflexed position, resulting in force being transmitted along the metatarsals longitudinally and resulting in compressive forces being applied to the TMT joints and Lisfranc ligament [1, 4]. An example of this is a person or weight falling onto the heel of a plantar-flexed foot of another player who is on the ground [4].

There is a three-stage classification system for low-energy Lisfranc injury or midfoot sprains coined by Nunley and Vertullo [11]. This classification is based on weight-bearing radiographs, measuring the degree of diastasis between the medial cuneiform and the base of the second metatarsus, with increased widening indicating higher-grade injuries [11, 12]. They hypothesized that the dorsal ligament, being the weakest, is the most commonly injured component, followed by injury of the interosseous Lisfranc ligament and then the plantar, as more substantial forces are applied. This classification usually implies an acute injury and does not include MRI findings [13]. Proximal variant patterns of instability have also been described, with transmission of force extending proximally through the intercuneiform joints and exiting via the navicular-cuneiform articulation [7, 14]. Whilst traditional teaching dictates that the dorsal ligament often gets injured first, the plantar component is reported to be more commonly injured in athletes [3, 15]. Our case reports reaffirm the observation.

When normal bone is subjected to abnormal or repetitive stress, it undergoes physiologic, adaptive changes with new bone deposition at the sites of the stress [16]. If the stress is sufficient enough to exceed the adaptive response, the bone weakens and becomes increasingly susceptible to injury and fatigue fracture ensues [17, 18]. MRI is superior to other imaging modalities in diagnosis, grading, and hence prognosticating bone stress injuries. On MRI, bone stress injuries often begin with periosteal oedema. This can progress to marrow oedema signal and, if untreated, cortical oedema and stress fracture [18]. Cortical thickening can be observed as part of the remodeling process. There are reports of stress fractures at the base of second metatarsals in high-level athletes which matches our cohort [19] although in the available literature, the Lisfranc ligaments were not specifically assessed.

We also note that stress fractures reported in ballet dancers characteristically occur at the base of the second metatarsals [19]. Whilst acknowledging that Lisfranc ligamentous injury is uncommon in ballet dancers given the en pointe position which imparts compressive rather than shearing force across the TMT joints [9, 20], there is paucity of literature on MRI correlate of ballet dancers with second metatarsal base fractures and Lisfranc ligaments in this cohort of patients. This would be an important area of research because the imaging of these second metatarsal base fractures described in literature are quite similar to our cases, involving the plantar Lisfranc footprints at metatarsal bases [9]. One may assume that chronic Lisfranc sprain could occur in ballet dancers if the technique is poor.

There is one case report in the English literature describing Lisfranc ligamentous injury in a low-energy context in the presence of bone stress reaction, as indicated by cortical reaction and thickening. However, this occurred spontaneously and did not occur in the context of a sports injury. The patient had a background of autoimmune condition (systemic lupus erythematosus and antiphospholipid syndrome) and osteoporosis. The presentation was also delayed, potentially confounding the imaging findings. Also, an MRI was not performed and Lisfranc ligamentous injury was only inferred based on the fracture which delineated the footprint of the Lisfranc ligament [10].

In our cases, the initial imaging was performed in a relatively short timeframe since the onset of symptoms, showing changes of bone stress response with Lisfranc ligamentous sprain. Extrapolating the available data on athletes and ballet dancers, it is expected that the Lisfranc ligament in this subset of high-performing athletes has to withstand a substantial amount of stress over a prolonged period of time, enough for bony adaptive response at the footprint of the Lisfranc ligaments with cortical remodeling and thickening. The symptoms may only manifest when this adaptive response fails, such as in the context of impending stress fracture. The authors would like to raise chronic stress response as a subset of low-energy injury to the Lisfranc ligament. This hypothesis will have to be further explored with prospective studies, potentially with longitudinal surveillance of these athletes.

Complications of untreated Lisfranc injuries include persistent pain, flattening of the longitudinal arch, and accelerated osteoarthritis in the midfoot. This injury is associated with significant morbidity with up to 18% of affected athletes being unable to return to baseline sports after injury [4]. The treatment goal is aimed at stable and painless midfoot and, hopefully, returning to play [3]. Early treatment and rest can prevent the progression to fracture and associated complication. Most patients can be treated nonoperatively with non-weight-bearing status or protected weight-bearing with boot or cast for 6 weeks, prior to progressive weight-bearing as tolerated [18]. This was the case in two of our athletes. The third athlete did not improve with conservative management and ultimately required internal fixation of the stress fracture, with good response thereafter.

In conclusion, low-energy injuries of the Lisfranc articulation, or midfoot sprain, are increasingly being recognised as a common sports injury, particularly in high-level athletes, with potential long-term morbidity. We present three cases in which the presentation and duration of symptoms of the high-level athletes do not fit with the degree of bone remodeling observed on MRI, suggesting sustained period of stress to the Lisfranc ligament in sporting activities. We speculate that there is a subset of chronic, repetitive, low-energy Lisfranc stress which can manifest as bone adaptive response and ligamentous sprain.

## Data Availability

Data is available on request due to privacy/ethical restrictions.
